# The complete chloroplast genome sequence of *Helicia shweliensis*

**DOI:** 10.1080/23802359.2019.1640086

**Published:** 2019-07-13

**Authors:** Yi Wang, Xiaolong Yuan, Ma Saiyu, Jinfeng Zhang

**Affiliations:** Laboratory of Forest Plant Cultivation and Utilization, Yunnan Academy of Forestry, Kunming Yunnan, People's Republic of China

**Keywords:** *Helicia shweliensis*, chloroplast, Illumina sequencing, phylogenetic analysis

## Abstract

*Helicia shweliensis* is a Critically Endangered species endemic to Yunnan, China. In this study, the complete chloroplast genome (cpDNA) sequence of *H. shweliensis* was determined from Illumina HiSeq pair-end sequencing data. The cpDNA is 157,151 bp in length, contains a large single copy region (LSC) of 85,463 bp and a small single copy region (SSC) of 18,662 bp, which were separated by a pair of inverted repeat (IR) regions of 26,513 bp. The genome contains 131 genes, including 86 protein-coding genes, 8 ribosomal RNA genes, and 37 transfer RNA genes. The overall GC content of the whole genome is 38.1% and the corresponding values of the LSC, SSC, and IR regions are 36.5, 31.8, and 42.9%, respectively. Further, phylogenomic analysis showed that *V. paradoxa* clustered in a unique clade in order Proteales.

*Helicia shweliensis* is the species of the genus Helicia within the family Proteaceae and whose natural distribution is restricted to China within the west of Yunnan (Ma et al. 2017). *Helicia shweliensis* is an important wild fruit tree resource (Yang et al. 2017) and *H. shweliensis* also is a Critically Endangered species endemic to Yunnan China (Gong 2006). However, there have been no genomic studies on *H. shweliensis*.

Herein, we reported and characterized the complete *H. shweliensis* plastid genome (MK962317). One *H. shweliensis* individual (specimen number: 2018040172) was collected from Gaoligong Mountains, Yunnan Province of China (24°50′26″ N, 98°45′15″ E). The specimen is stored at Yunnan Academy of Forestry Herbarium. DNA was extracted from its fresh leaves using DNA Plantzol Reagent (Invitrogen, Carlsbad, CA, USA).

Paired-end reads were sequenced by using Illumina HiSeq system (Illumina, San Diego, CA, USA). In total, about 43.5 million high-quality clean reads were generated with adaptors trimmed. Aligning, assembly, and annotation were conducted by CLC de novo assembler (CLC Bio, Aarhus, Denmark), BLAST, GeSeq (Tillich et al. 2017), and GENEIOUS v 11.0.5 (Biomatters Ltd, Auckland, New Zealand). To confirm the phylogenetic position of *H. shweliensis*, other seven species of order Proteales from NCBI were aligned using MAFFT v.7 (Katoh and Standley 2013) and maximum likelihood (ML) bootstrap analysis was conducted using RAxML (Stamatakis 2006); bootstrap probability values were calculated from 1000 replicates. *Sideroxylon wightianum* (MG719834) was served as the outgroup.

The complete *H. shweliensis* plastid genome is a circular DNA molecule with the length of 157,151 bp, contains a large single copy region (LSC) of 85,463 bp and a small single copy region (SSC) of 18,662 bp, which were separated by a pair of inverted repeat (IR) regions of 26,513 bp. The overall GC content of the whole genome is 38.1% and the corresponding values of the LSC, SSC, and IR regions are 36.5, 31.8, and 42.9%, respectively. The genome contains 131 genes, including 86 protein-coding genes, 8 ribosomal RNA genes, and 37 transfer RNA genes. Phylogenetic analysis showed that *H. shweliensis* clustered in a unique clade in order Proteales (Figure 1). The determination of the complete plastid genome sequences provided new molecular data to illuminate the Proteales evolution.

**Figure 1.**
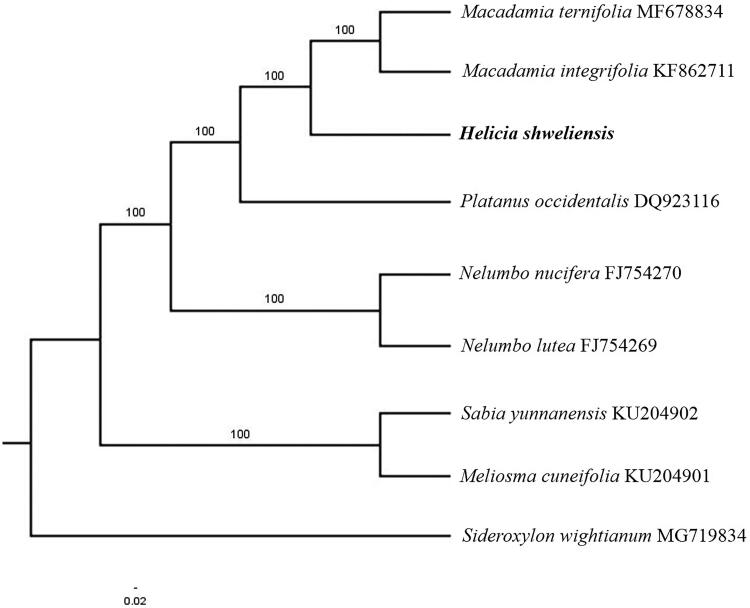
The maximum-likelihood tree based on the 8 chloroplast genomes of order Proteales. The bootstrap value based on 1000 replicates is shown on each node.
